# Patterns of Food Consumption of a Sample of College Students Using Superseded Canada’s Food Guide

**DOI:** 10.3390/foods15183307

**Published:** 2026-09-18

**Authors:** Mohammed H. Moghadasian, Hibah Khawar

**Affiliations:** 1 Department of Food and Human Nutritional Sciences, University of Manitoba, Winnipeg, MB R3T 2U2, Canada; 2 Canadian Centre for Agri-Food Research in Health and Medicine, St. Boniface Hospital Albrechtsen Research Centre, Winnipeg, MB R2H 2A6, Canada

**Keywords:** food consumption, food groups, fruits and vegetables, plant-based foods, nutrients, dietary fiber, college student

## Abstract

Our previous studies using large national datasets revealed that most Canadian adolescents and elderly people do not consume adequate amounts of fruits and vegetables. The present study aimed to explore the patterns of food consumption in a sample of college students. This is a secondary analysis of a master’s thesis completed in 2018. Sixty-eight students completed the study by providing a 3-day food record. Considering the 2011 Canada’s Food Guide, the consumption of four main food groups, plus the intakes of energy, nutrients, dietary fiber, and water, were estimated. The self-identified female and male participants had different food consumption profiles for all food groups; this variation was more prominent in fruits and vegetables as well as milk and dairy foods. The consumption of plant-based foods was below the recommended amounts in most of the students. The intakes of energy, proteins, fats, cholesterol, vitamins (A, C, D), sodium, and dietary fiber significantly varied between the two groups of students. Additional studies are needed to establish whether these findings may be applicable to larger adult populations.

## 1. Introduction

Dietary recommendations are meant to support physiologic growth and development as well as to prevent or postpone the onset of non-communicable chronic diseases. We have previously reported that Canadian adolescents and elderly people do not adequately consume fruits and vegetables [1,2]. Significant changes in lifestyle, including food consumption, take place during early adulthood. Various factors such as job, education, travel, marital status and other family-related matters, diseases, and inflation influence access to high-quality food and food consumption. In particular, college students may experience a high degree of vulnerability to inadequate food consumption due to them starting to live alone and experiencing independence in managing their day-to-day life events, including earning an income to be able to purchase high-quality foods as well as cooking, cleaning, and general self-care responsibilities. These matters could be very challenging and may negatively impact food consumption and nutrient intake.

University/college students are expected to follow dietary guidelines and, thereby, consume adequate amounts of high-quality foods. However, personal, social, financial, and school-related factors may compromise their food consumption. Previous studies have reported that skipping breakfast and consuming fast foods, highly processed foods, and sugar-sweetened beverages are common eating behaviors among university students [3,4,5,6]. Lifestyle and dietary behavior may contribute to the established sex-related variations in quality of life and life expectancy. Overall, morbidity and mortality rates vary significantly between women and men [7,8,9]. A 2026 study of 47,056 American adults reported that after adjusting for all of the confounding factors, the risk for all-cause mortality was 63% higher in men than in women [10]. We have shown that, in general, self-identified female individuals follow a healthier lifestyle and dietary behavior than their self-identified male counterparts [1,2]. Studies of sex and gender differences in health and disease have been reviewed and discussed elsewhere [7,8,9,11].

This study is a secondary analysis of data obtained from 68 students’ assignments for the completion of an introductory Nutrition course offered in 2015 and 2016. The students identified themselves on their food records as “male” or “female”; thus, the investigators could not verify whether such self-identification represents biological sex or socio-cultural gender [11]. At the time of this investigation, the investigators noticed a gap in knowledge about the patterns of food consumption among the college students studying nutrition and how their nutrition education may impact their adherence to dietary guidelines. Another knowledge gap in this field was the extent to which such patterns of food consumption influence their overall nutrient intakes. Thus, the present research served as an exploratory small-scale study to investigate the patterns of food consumption (Primary Objective) and nutrient intake (Secondary Objective) among such students, using the 2011 Canada’s Food Guide (CFG) [12]; this version of CFG was in effect during the study period of 2015 and 2016. The Tertiary Objective of this investigation was to learn about other aspects of lifestyle that may be directly or indirectly associated with the patterns of food consumption. These included body mass index (BMI), physical activity levels, and cigarette smoking. This study contributes to our understanding of how closely college students may follow dietary guidelines. The findings of this study suggest that additional efforts are needed to promote dietary guidelines among all segments of the general population. The data from the present small sample of college students clearly show that even nutrition students—despite their educational topic of interest—may not follow the national dietary guidelines. Furthermore, the less-than-optimal consumption of various food groups was accompanied by less-than-optimal reported physical activity levels. The present study was not designed to investigate the roots of this potentially “unhealthy” eating behavior and inactivity among most of the students. The findings of this small-scale study may not be applicable to larger adult populations. Therefore, additional studies are needed to verify or disapprove the applicability of our results to adult populations. The present report has been produced from a 2018 MSc thesis [13].

## 2. Materials and Methods

### 2.1. Study Population and Data Collection

The participants in this cross-sectional convenience sampling study were university students who took an introductory nutrition course in the summers of 2015 and 2016. The participants were trained to prepare and submit their 3-day food records (2 consecutive weekdays and 1 weekend day), reporting their food consumption in the form of four food groups: fruits and vegetables (F&V), meat and alternatives (M&A), milk and alternatives (Mk&A), and grain products per the 2011 CFG [12]. It should be mentioned that the 2011 CFG used to recommend the consumption of the 4 food groups, including V&F, M&A, Mk&A and grain products as the *Recommended Number of Food Guide Servings per Day* for each age and sex group, including children (2–3 years, 4–8 years and 9–3 years); teens (14–18 years) and adults (19–50 and 51+ years); the recommended number of servings varied for females and males older than 14 years [12]. The 2019 CFG does not recommend a number of servings, nor does it distinguish the four classes of foods; instead, the 2019 CFG is a plate-based guide, recommending half of the plate from F&V, ^1^/_4_ of the plate protein foods, and the last ^1^/_4_ of the plate grain products with an emphasis on whole grain products [14]. As can be understood, the principle of both versions of the CFG is to include most of the daily food intake from F&V and approximately similar amounts of protein foods (meats, fish, eggs, dairy) and grain and starchy food products, altogether counting for the other half of the plate. The 2019 version of the CFG is more consumer-friendly and easier to follow because consumers do not need to measure/estimate their food serving sizes. The students learned how to use CFG principles, including measuring/estimating portion sizes using various means/tools, such as cups, tablespoons, teaspoons, palm of hand, etc.

Food Focus software version 4.1 was used to analyze the food consumed and, accordingly, estimate nutrient intakes; this version provided analytical information for 7278 food items. The students were given instructions to adequately describe their diet, including the quantity of foods and drinks consumed and their major characteristics (e.g., color: green or yellow beans, white or whole wheat bread; freshness: fresh, frozen, canned or dried; fat content: % fat content of dairy products, i.e., 1%, 2% or homo milk or the leanness of meat such as extra lean ground beef). For home-prepared or mixed dishes, Food Focus allowed the user to enter the amount of each raw ingredient plus the method of cooking (barbecued, fried, boiled, etc.). The packaged food items were reported in package size, for example: 15 mL of frozen yogurt. Details about restaurant food, fast food, or packaged food items required the name of the brand, such as McDonald’s Big Mac or KFC Zinger. The software allowed users to add multiple days. Once all food items were entered, the software estimated the nutrient content of each food item, yielding information on energy and nutrient intakes. For food groups, students were required to segregate the food items into 4 food group categories: F&V, grain products, Mk&A, and M&A. For mixed dishes such as salads, food items were segregated into their major ingredients to be classified into each food group. The above information was obtained for the original thesis [13].

The students also provided their age, height, weight, smoking habits, physical activity levels, and the daily cost of their meals; the students identified themselves as male or female. An independent third party removed all identifying marks permanently from these food records. Therefore, the investigators performed their analyses of data in a blinded fashion.

### 2.2. Statement of the Ethics Approval and Informed Consent

The present paper reports the secondary use of the original food records submitted by the students for the completion of their introductory nutrition course. This paper is a completely modified form of an MSc thesis completed in 2018 [13]. Furthermore, this paper has been produced in accordance with our previous publications created through the secondary use of nationally produced original data [1,2,15]. We have followed the principles of Tri-Council Policy Statement Ethical Conduct for Research Involving Humans (TCPS2 2022) [16]. Such principles, as stated on Pages 29, 54, and 270 of the TCPS2 [16], recognize the nature of this research as an educational and training activity; therefore, the participation of the students was required for the completion of the course. Furthermore, this research activity involved no more than minimal risk to the participants; likewise, a lack of informed consent requirement was unlikely to adversely affect the welfare of the participants. Additionally, due to the nature of the course assignment/design, the research objective could not be achieved if informed consent was sought prior to the initiation of the course. Therefore, this training activity did not require obtaining direct informed consent from the students. Furthermore, this research was completed under the anonymized category. Under this category, all identifying marks were removed irrevocably by an independent third party. This process made any future re-linkage and the risk of re-identification of the participants almost impossible. The experts within the Advisory Committee of the MSc thesis approved this investigation under the above-mentioned conditions and allowed the graduate student to submit her thesis [13].

### 2.3. Inclusion and Exclusion Criteria

Inclusion criteria were (a) enrollment in the course; (b) completion of all parts of the assignments; and (c) self-identification as “male or female”. Exclusion criteria were (a) age under 19 or over 50; (b) pregnancy and lactation; and (c) incomplete assignments. A boxplot and scatter plot were used to identify the outliers [17,18]. This analysis resulted in the exclusion of the following: lacking appropriate data entry (n = 8), incomplete data information (n = 6), and errors with mixed dishes (n = 14), as reported in the original thesis [13]. Some of these errors in data entry or coding included reporting, for example, intakes of 72,166 mg/day for sodium, 763 mg/day for iron, and 8736 kcal/day for energy. Other reasons for exclusion included pregnancy (n = 1), age below 19 years (n = 5), and incomplete and missing food logs (n = 6). We were confident that the excluded food records were not suitable to contribute to the objectives of our study; this matter was also approved by the expert Advisory Committee of the MSc thesis [13].

Of the 108 participants, 40 were excluded, and 68 (44 self-identified females and 24 self-identified males) completed the study. We were unable to distinguish the self-identified “male” or “female” participants with regard to the “biological sex” or “socio-cultural gender” concepts of males or females. Therefore, in this article, the participants are identified as “male” or “female” per their original statements.

### 2.4. Data Mining: Normality Test and Missing Values

The “listwise deletion” procedure [19] was utilized for the exclusion of data if lacking one of the variables of interest. To assess the normality of the data sets, we used visual inspection using a *Q-Q* plot and boxplot, as well as Kolmogorov–Smirnov normality tests (K-S test) and a *z*-test. The normality of distribution for variables was also numerically tested, using skewness and kurtosis values [20] to determine the symmetry of the dataset and the concentration of data around the mean, respectively [17]. The absolute z-value was calculated using skewness and kurtosis values [17,19]. Details of these procedures are available in the original thesis [13].

### 2.5. Evaluating the Number of Servings from Four Food Groups per 2011 CFG

The participants were guided to estimate the number of servings from each of the four food groups using common tools such as cups and spoons, dimensional units, or weight, as appropriate to the food item consumed. The estimated number of servings for each food group was recorded as a number, such as 0.5, 1, or 1.5. To evaluate the adequacy of intake, the estimated number of servings for each of the four food groups was compared with the 2011 CFG recommended number of servings for 19–50-year-old males or females. An intake at or above the 2011 CFG number of servings for self-identified males or self-identified females was considered adequate [12,13].

### 2.6. Calculation of Energy Expenditure Requirement (EER)

Standard equations were used to calculate EER for the self-identified male and self-identified female participants. The details of the calculations and the formulas are available in the original thesis [13].

### 2.7. Estimation of Macronutrient and Micronutrient Intakes

Food Focus software version 4.1 was used to estimate the intakes of both macronutrients and micronutrients. Details on the application and other information are available in the original thesis [13].

### 2.8. Statistical Analysis

The Statistical Package for the Social Sciences (SPSS) version 24.0 for Windows was used. Continuous variables were presented as means with standard errors plus medians with interquartile ranges (IQR), whereas the categorical variables were expressed as frequencies (%). An independent *t*-test or Mann–Whitney U test was used to compare the mean intake of each self-identified sex group. Statistically significant differences were considered at *p* < 0.05. Details are available elsewhere [13].

## 3. Results

### 3.1. Demographic Characteristics

**Figure 1:**
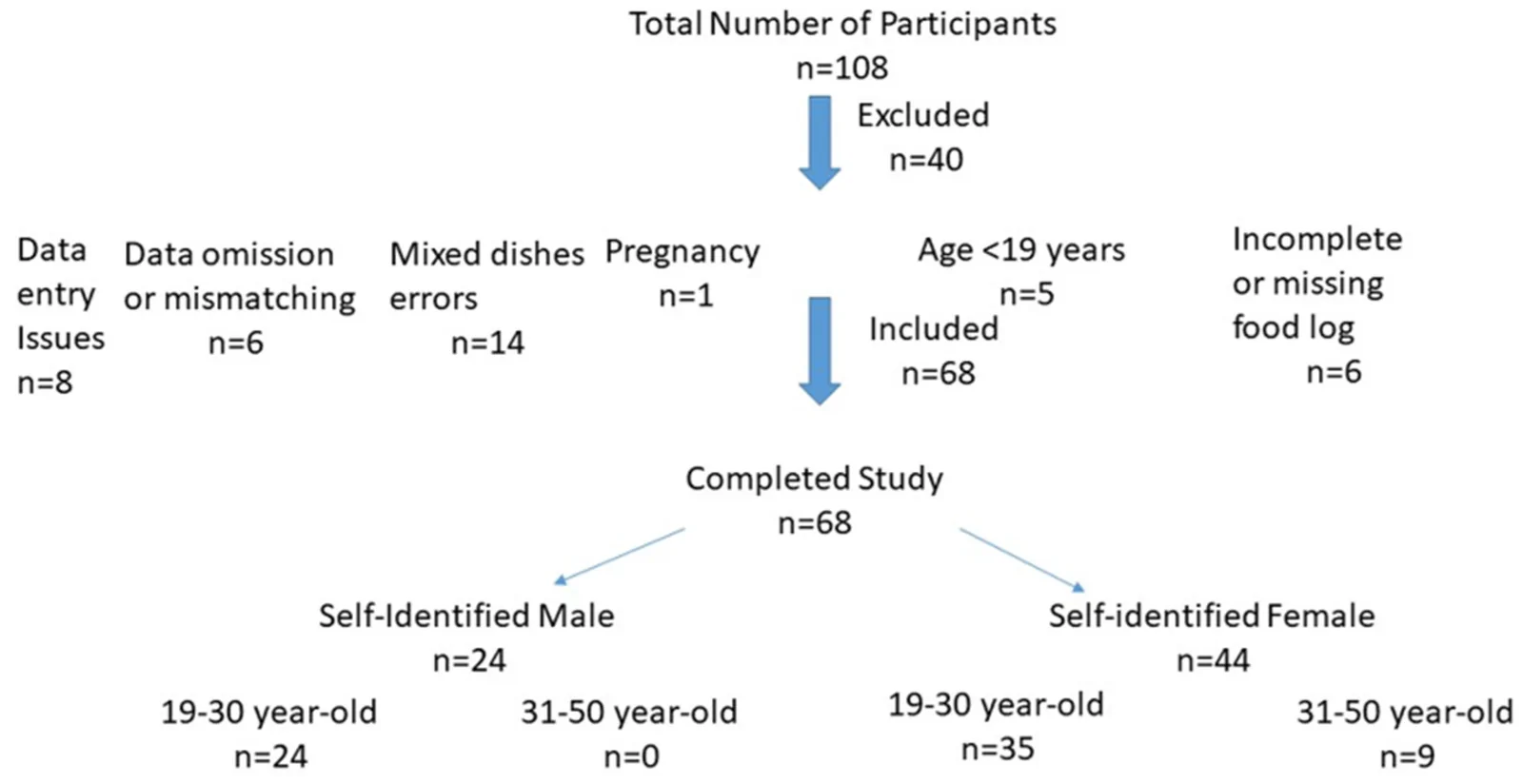
Study Participant Flowchart. Flowchart representing the study population. Out of 108 participants, a total of 24 self-identified males and 44 self-identified females completed the study.

As shown in Figure 1, 108 students submitted their assignments; however, only 68 students’ assignments from 44 self-identified females and 24 self-identified males were included in this analysis. Forty assignments were excluded for the following reasons: (a) pregnancy (1 student); (b) age below 19 years (5 students); (c) incomplete and missing food log (6 students); (d) data entry issues (8 students); (e) data omission and mismatching (6 students); and (f) errors in mixed dishes (14 students). The demographic information of the participants is summarized in Table 1. Twenty-four (35%) self-identified male and 44 (65%) self-identified female students completed the study. The mean age of the self-identified males and self-identified females was comparable. All of the participants were 19–30 years old, except for 9 self-identified females who were 31–50 years old. BMI analysis revealed that 57% of the participants were in the normal weight category (Table 1). The prevalence of cigarette smoking was significantly higher in the self-identified male participants than in the self-identified female participants (Table 1).

### 3.2. Food Group Consumption

**Figure 2:**
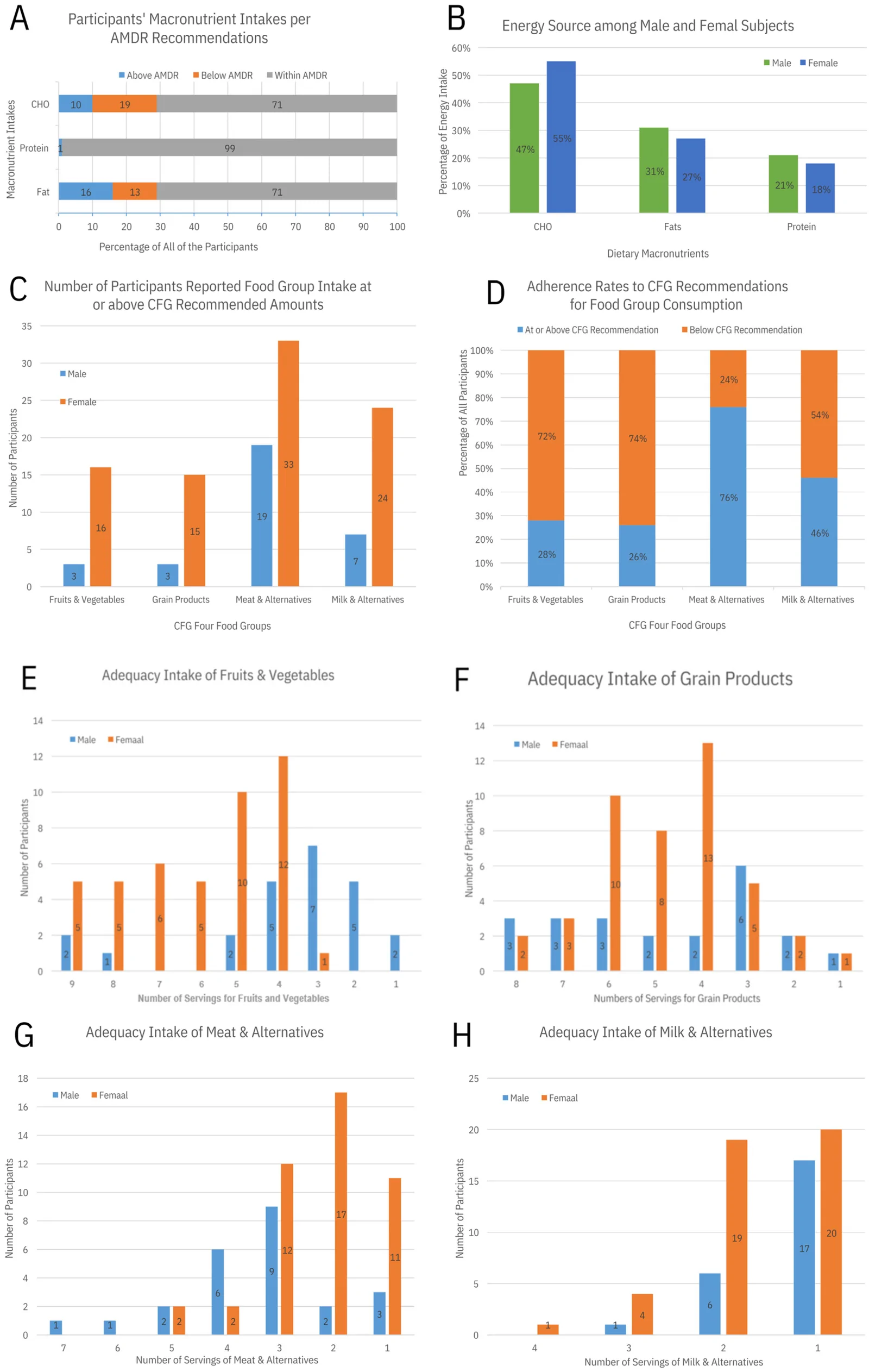
Food and Energy Consumption. The patterns of energy-yielding nutrients and the consumption of the four food groups among all of the participants, per the 2011 Canada’s Food Guide. Panel (**A**) shows the percentage of participants who consumed the energy-yielding nutrients below, within, and above the Adequate Macronutrient Distribution Range (AMDR) recommendations. Panel (**B**) exhibits the percentage of energy intake from macronutrients among the participants. Panel (**C**) depicts the number of self-identified female and self-identified male subjects who reported consuming servings for each of the four food groups at or above the 2011 Canada’s Food Guide recommended amounts. Panel (**D**) presents the percentage of self-identified female and self-identified male individuals who followed or did not follow the 2011 Canada’s Food Guide on the consumption of each of the four food groups. Panels (**E**–**H**) illustrate the frequency of the number of servings of fruits and vegetables, grain products, milk and alternatives, and meat and alternatives, respectively, consumed by the participants. **Note:** At the time of this investigation, Canada’s Food Guide recommendations for 19–50-year-olds were the following: **Vegetables and Fruits:** 7–8 servings for females and 8–10 servings for males, **Grain Products:** 6–7 servings for females and 8 servings for males, **Milk and Alternatives:** 2 servings for both females and males, **Meat and Alternatives:** 2 servings for females and 3 servings for males.

Data regarding food consumption per the 2011 CFG recommended number of servings, as well as the intakes of macronutrients and the source of energy intake, have been illustrated in Figure 2. Panel A demonstrates that 71% of the participants reported their carbohydrate and fat intakes to be within the acceptable macronutrient distribution range (AMDR) recommendations; this value was 99% for protein consumption. Panel B shows that most of the energy intake was from carbohydrates, followed by fats and proteins. Panel C illustrates that only 3 out of 24 self-identified males and 16 out of 44 self-identified females reported adequate intakes of F&V. These rates were almost the same for the consumption of grain products. In contrast, 79% of the self-identified males and 75% of the self-identified females consumed M&A adequately. Seven out of 24 self-identified males and 24 out of 44 self-identified females consumed an adequate number of servings of Mk&A. Panel D breaks down the percentage of all participants with or without adherence to the 2011 CFG recommendations [12] for the consumption of the four food groups. Overall, more than 70% of the participants did not consume adequate amounts of plant-based foods. The percentage of the participants who consumed adequate amounts of M&A or Mk&A was 76% or 46%, respectively. The frequency of the estimated number of servings for each food group is depicted on Panels E–H. Panel E shows that 3 servings of F&V were the most frequent intake among the self-identified men, while 4 servings were the most frequent among the self-identified women. Panel F demonstrates that the most frequent number of servings for grain products was 4 for the self-identified women and 3 for the self-identified men. The most frequent intakes of M&A were 3 and 2 servings among the self-identified males and self-identified females, respectively (Panel G). Panel H shows that 19 self-identified females and 6 self-identified males reported complete adherence to the 2011 CFG recommendations for the consumption of Mk&A.

### 3.3. Nutrient Intakes

**Figure 3:**
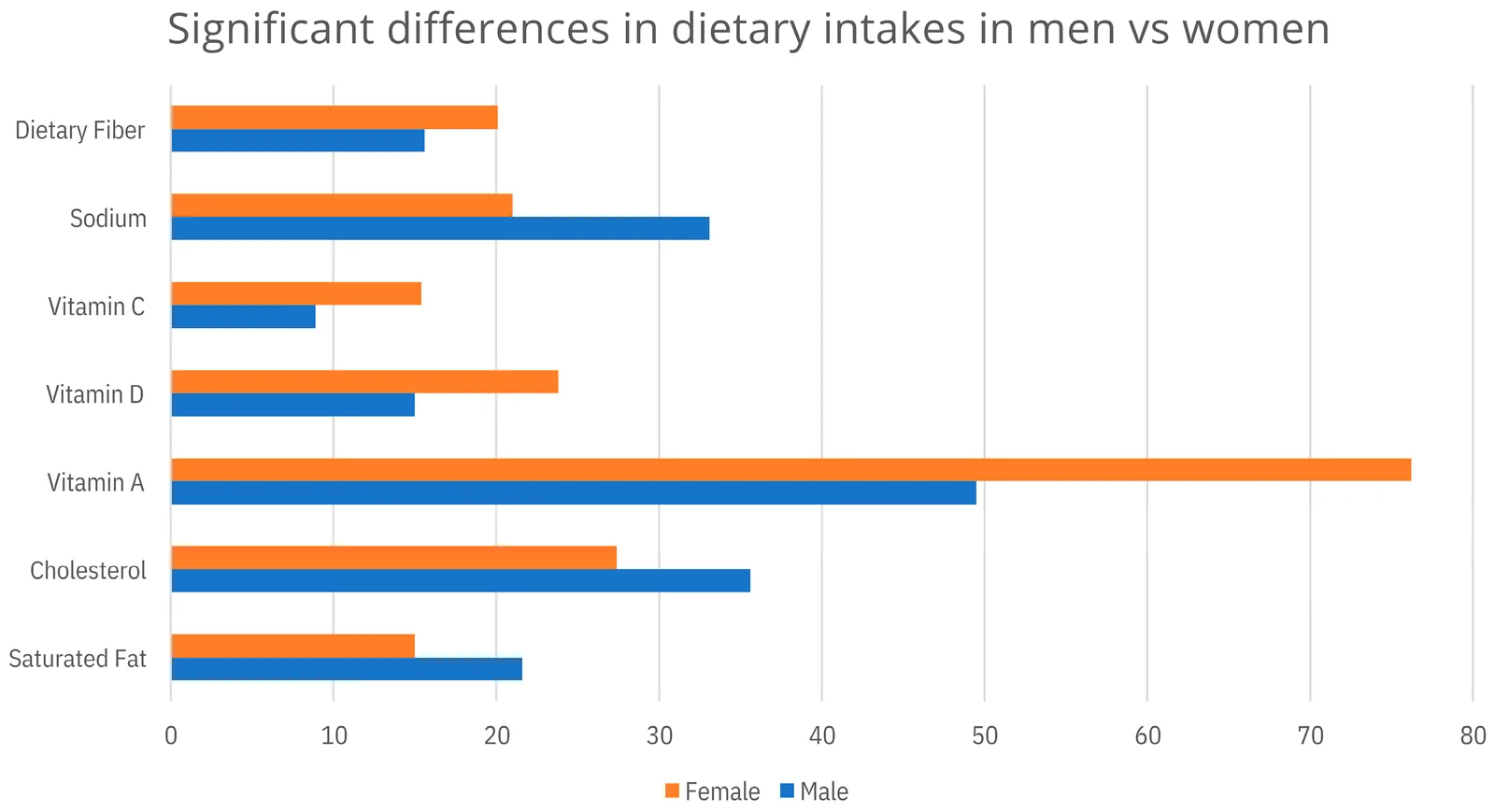
Nutrient Intake Differences between the two groups. Significant (*p* < 0.05) differences in daily dietary intakes reported by self-identified male and self-identified female students. Units Descriptions: Dietary Fiber (grams); Sodium (milligrams × 100); Vitamin C (milligrams × 10); Vitamin D (International Units × 10); Vitamin A (micrograms × 10); Cholesterol (milligrams × 10); Saturated Fat (grams).

Table 2 summarizes the estimated nutrient intakes of the participants. The intakes of some nutrients were significantly different between the two groups of students. These consisted of variations in intakes of energy, protein, total fat, saturated fat, monounsaturated fatty acids (MUFA), cholesterol, vitamins A, C, and D, sodium, and dietary fiber (Table 2). Figure 3 summarizes the large gaps between the two groups of participants with regard to select nutrient intakes. For example, the self-identified male subjects reported significantly (*p* < 0.05) lower intakes of dietary fiber, vitamin C, vitamin D, and vitamin A than their self-identified female counterparts. On the other hand, the self-reported male students reported significantly (*p* < 0.05) higher intakes of sodium, cholesterol, and saturated fat as compared to the intakes of the self-identified female students.

Table 3 shows that the reported energy intake was less than the calculated EER for both groups of students. The reported energy intake did not seem to be consistent with the reported physical activity levels. As shown in Table 3, the calculated EI/EER ratio for the subgroups of students by their physical activity levels ranged from 0.53 to 0.88 for the self-identified females and from 0.68 to 0.81 for the self-identified males.

## 4. Discussion

We have used quantitative approaches to investigate the patterns of consumption of the four groups of foods among university students taking an introductory nutrition course using the 2011 CFG. Previous studies highlighted that nutrition education plays an important role in shifting minds towards healthy eating behaviors [21,22,23]. However, other studies reported that university students face numerous challenges that may negatively impact their food selection and healthy eating behaviors [24,25,26]. Our current data clearly demonstrate that despite being interested in studying nutrition, some of the participants did not follow the 2011 CFG recommendations [12]. At the time of this investigation, CFG recommended an age- and sex-specific number of servings for each of the four food groups, consisting of plant-based (F&V and grain products) and animal-based (Mk&A and M&A) foods. Our data show that more than 70% of the participants did not meet the recommendations for plant-based food consumption (Figure 2). Both short- and long-term observational and epidemiological investigations suggest a meaningful relationship between the less-than-optimal consumption of various food groups and the state of health. A 2021 review and meta-analysis of data from 26 cohort studies, including a total of 145,015 deaths among 1,892,885 participants, reported a significant association between total mortality rates and the amount of F&V consumed [27]. Results from this review showed that the consumption of 5 servings of F&V significantly reduced the risk of total mortality. In particular, when compared with 2 servings, 5 servings of F&V per day resulted in hazard ratios of 0.87, 0.88, 0.90, and 0.65 for total mortality, cardiovascular mortality, cancer mortality, and respiratory disease mortality, respectively. It should be mentioned that such an association was not observed for starchy vegetables like corn, potatoes, and peas, nor for fruit juices. This study suggests that adherence to the recommendations for the consumption of F&V will lead to reduced total and cause-specific mortality; however, this benefit plateaued at 5 servings per day [27]. Our data show that 79% of self-identified male and 29% of self-identified female students consumed less than 5 servings of F&V per day according to the 2011 CFG. It should be mentioned that the current CFG does not recommend any specific number of servings for food groups; however, it recommends that half of the plate for each meal should be F&V [14].

Another review of 3 prospective cohort studies concluded that higher consumption of whole-grain food products was significantly associated with reduced risk of type 2 diabetes [28]. These findings suggest that adherence to dietary recommendations for intakes of whole-grain food products may be an effective way to prevent or postpone the onset of type 2 diabetes, though the 2011 CFG did not specifically recommend whole-grain products. More than 50% of our participants did not consume adequate amounts of grain products.

Plant-based foods are the source of dietary fiber, and our data suggest that the consumption of dietary fiber may be less than optimal in some of the participants. A recent clinical study reported that the consumption of dietary fiber was associated with lower inflammation and risk of cardiovascular disease among American older adults [29]. A pooled analysis of data from 1,445,850 women and men revealed that the combination of dietary fiber with yogurt was associated with reduced risk of lung cancer [30]. Furthermore, epidemiological and observational studies have suggested gastrointestinal health benefits of dietary fiber through the consumption of unrefined whole foods such as whole-grain products, legumes, or F&V [31].

The pattern of consumption of animal-based foods was less concerning among our students, as only 24% and 54% of the participants did not consume adequate amounts of M&A or Mk&A per the 2011 CFG, respectively (Figure 2). A meta-analysis study reported that lower intakes of red meat were associated with lower risk of colorectal cancer [32]. On the other hand, a cohort study of 1011 stage III colon cancer patients found that the consumption of unprocessed red meat or processed meat did not increase the risk of recurrence or mortality among those patients [33]. A sex-related association was found between mortality and meat consumption among the NHANES III participants [34].

Approximately half of our participants did not consume adequate amounts of milk and other dairy products (Figure 2). Milk and other dairy products are rich sources of many high-quality nutrients, including calcium, vitamins D, A, and B12, essential amino acids, zinc, magnesium, and potassium [35]. Furthermore, these products contain other biologically active ingredients such as bioactive peptides and cultures with health-promoting properties as well as water [36]. Of the many health benefits of milk and alternatives, their effects on bone health have been long recognized [37].

The estimated energy intake was lower than the EER values (Table 3); this could be related to a common phenomenon of underreporting energy intake. Bedard et al. [38] investigated the state of underreported energy intake among 246 Canadian adults by administering a semi-quantitative food-frequency questionnaire (FFQ). The results showed that 54% of male and 35% of female participants underreported their energy intake according to the Goldberg statistical cut-off EI/BMR method [39]. Similarly, Yanez and colleagues [40] reported that among Health Sciences students, 44% (at group level) of the students underreported their energy intake. A review of 22 studies reported that, on average, 34% of study subjects may underreport their energy intake [41]. This study also reported that physical activity level is a major determinant of the sensitivity and specificity of the Golberg/Black EI/BMR ratio method. In this regard, the reported energy intake of our students was not proportionate to their levels of physical activity. This possibly underreported energy intake was associated with underweight (BMI < 18.5) status rates of 8% and 18% among the self-identified male and self-identified female students, respectively (Table 1).

Approximately 35% of the participants reported an active lifestyle, and 65% reported either a sedentary or low activity status (Table 3). A review of 21 international studies, including 7306 male and female students, reported that many factors contribute to the level of physical activity. These factors include differences in culture and education systems of various countries [42]. A healthy lifestyle, including maintaining a healthy body weight and performing regular physical activities, is essential for optimal health and productivity. Indeed, a recent retrospective analysis of a UK Biobank cohort study involving 89,573 women and men reported a negative association between the pattern of physical activity and the incidence of atrial fibrillation, myocardial infarction, heart failure, and stroke [43]. Another study highlighted that university students are vulnerable to mental disorders due to high sitting times and overall low physical activity. This study suggested that regular exercise at low to moderate intensity may improve the mental health of university students [44]. It is due to numerous health benefits that national and international guidelines on physical activity have been developed [45,46,47,48]. Furthermore, the consequences of physical inactivity and sedentary behavior, plus a state of undernutrition, on obesity and cardiovascular health have been discussed elsewhere [49].

The limitation of this study is the self-reported data and the investigators’ inability to cross-check; this is a common limitation of all investigations on the analysis of self-reported data. Other limitations may include a relatively small sample size. However, taking into account that our data are in agreement with data reported from much larger studies may endorse the validity of our data, despite coming from a relatively small group of college students. For example, we reported [1,2] that thousands of female and male subjects participated in national government surveys and reported similar dietary behavior, particularly in regard to plant-based food consumption. The 3-day food record method, like several other dietary intake assessment methods, has limitations. One such limitation could be related to social desirability bias, particularly in a multicultural society like Canada. Among other limitations, potential confounding factors such as living arrangements, cooking skills, and meal planning can be named. Another limitation of this study could be the fact that the recruitment of students from an introductory nutrition course may indicate a self-selected sample with higher nutritional literacy, compromising external validity.

In conclusion, this study reports the patterns of food consumption among a sample of college students using the 2011 CFG. This small sample of self-identified male and female students shows significant variations with regard to food consumption plus other aspects of lifestyle such as levels of physical activity, BMI values, and cigarette smoking. The consumption of plant-based foods seemed inadequate in both groups of students. This was associated with a probably underreported intake of energy, calcium, vitamin D, and dietary fiber. Following dietary guidelines will ensure optimal health and productivity. Increased awareness in the general population about the importance of dietary behaviors and adherence to the national dietary guidelines can be achieved through various means such as local media, social media, special events, and others.

## Figures and Tables

**Table 1:** Demographic characteristics of the participants.

		All (n = 68)	Self-Identified Male (n = 24)	Self-Identified Female (n = 44)	*p*-Value
Age (year)	All Participants	23.7 (0.7)	21.8 (0.4)	24.8 (1.1)	0.42
	19–30 years	59 (87%)	24 (100%)	35 (79.5%)	
	31–50 years	9 (13%)	0 (0%)	9 (20.5%)	
Anthropometric	Height (m)	1.68 (0.01)	1.74 (0.01)	1.64 (0.01)	<0.01
	Weight (kg)	65.1 (1.6)	70.8 (2.0)	62.0 (2.0)	<0.01
	BMI (kg/m^2^)	23.1 (0.5)	23.3 (0.7)	22.9 (0.7)	0.39
	BMI < 18.5	10 (15%)	2 (8%)	8 (18%)	
	BMI 18.5–24.9	39 (57%)	14 (58%)	25 (57%)	
	BMI 25.0–29.9	14 (21%)	7 (29%)	7 (15%)	
	BMI > 30	5 (7%)	1 (4%)	4 (9%)	
Cigarette Smoking		6 (9%)	5 (21%)	1 (2%)	<0.05
Cost of Meal Per Day ($CAD)		7.9 (0.4)	9.8 (0.6)	6.9 (0.5)	<0.05

Data are the mean and Standard Error of the mean. *p*-values show statistically significant differences between the 2 groups of the participants.

**Table 2:** Estimated energy and nutrient intakes of the participants.

Intakes	Subjects	Mean (SE)	95% CI	Median (IQR)	*p*-Value
Energy (kcal/day)	All (n = 68)	1789 (62)	1664–1914	1682 (734)	0.012
	Male (n = 24)	1990 (109)	1764–2216	1982 (770)	
	Female (n = 44)	1679 (71)	1535–1824	1640 (542)	
CHO (g/day)	All (n = 68)	234 (9.1)	216–252	227.9 (93.6)	0.998
	Male (n = 24)	234 (16.6)	199–268	229.9 (116.6)	
	Female (n = 44)	234 (10.9)	212–256	227.9 (87.8)	
Total Sugar (g/day)	All (n = 68)	72.6 (4.3)	64.0–81.2	66.5 (41.8)	0.089
	Male (n = 24)	63.6 (6.4)	50.4–76.9	58.3 (35.7)	
	Female (n = 44)	78.5 (5.5)	67.2–89.8	71.7 (36.6)	
Added Sugar (g/day)	All (n = 68)	30.8 (3.4)	24.0–37.5	23.8 (23)	0.773
	Male (n = 24)	30.8 (6.1)	18.1–43.6	24.1 (25)	
	Female (n = 44)	30.7 (4.0)	22.7–38.7	23.7 (23)	
Proteins (g/kg BW/day)	All (n = 68)	1.3 (0.1)	1.2–1.4	1.2 (0.7)	0.036
	Male (n = 24)	1.5 (0.1)	1.3–1.6	1.5 (0.6)	
	Female (n = 44)	1.2 (0.1)	1.1–1.4	1.2 (0.7)	
Fats (g/day)	All (n = 68)	57.5 (2.9)	51.7–63.2	54.8 (33.6)	0.006
	Male (n = 24)	68.0 (5.1)	57.5–78.4	64.2 (30.5)	
	Female (n = 44)	51.7 (3.2)	45.3–58.2	48.3 (28.1)	
Saturated Fats (g/day)	All (n = 68)	17.3 (1.0)	15.3–19.4	16.2 (9.3)	0.001
	Male (n = 24)	21.6 (2.0)	17.5–25.7	18.6 (6.9)	
	Female (n = 44)	15.0 (1.0)	12.9–17.1	13.9 (8.5)	
Trans Fats (g/day)	All (n = 68)	0.5 (0.1)	0.4–0.7	0.4 (0.7)	0.195
	Male (n = 24)	0.6 (0.1)	0.4–0.7	0.6 (0.7)	
	Female (n = 44)	0.5 (0.1)	0.3–0.8	0.3 (0.6)	
MUFA (g/day)	All (n = 68)	22.5 (1.3)	20.0–25.1	20.1 (11.0)	0.002
	Male (n = 24)	27.7 (2.0)	23.5–31.9	24.7 (15.8)	
	Female (n = 44)	19.7 (1.5)	16.6–22.7	19.4 (10.7)	
Linoleic Acid (g/day)	All (n = 68)	8.1 (0.6)	6.8–9.4	8.3 (5.5)	0.249
	Male (n = 24)	9.3 (1.0)	7.0–11.6	9.0 (5.0)	
	Female (n = 44)	7.7 (0.8)	6.0–9.3	8.3 (5.5)	
Alpha-linolenic Acid (g/day)	All (n = 68)	1.4 (0.3)	0.8–2.1	0.9 (0.8)	0.983
	Male (n = 24)	1.2 (0.2)	0.6–1.7	0.9 (0.7)	
	Female (n = 44)	1.5 (0.4)	0.6–2.4	1.0 (0.9)	
EPA + DHA (g/day)	All (n = 68)	0.3 (0.1)	0.1–0.5	0.1 (0.2)	0.549
	Male (n = 24)	0.2 (0.1)	0–0.6	0.0 (0.6)	
	Female (n = 44)	0.3 (0.1)	0.1–0.5	0.1 (0.2)	
Cholesterol (mg/day)	All (n = 68)	303 (20)	263–343	268 (204)	0.036
	Male (n = 24)	356 (38)	277–436	283 (176)	
	Female (n = 44)	274 (22)	229–319	239 (186)	
Vitamin A (ug/day)	All (n = 68)	677 (45)	586–767	601 (443)	0.004
	Male (n = 24)	495 (68)	354–636	422 (509)	
	Female (n = 44)	762 (54)	654–870	649 (475)	
Vitamin D (IU/day)	All (n = 68)	200 (20)	160–239	154 (169)	0.028
	Male (n = 24)	150 (26)	96–204	104 (126)	
	Female (n = 44)	238 (28)	181–295	166 (206)	
Vitamin E (mg/day)	All (n = 68)	7.0 (0.5)	6.1–8.0	5.6 (4.7)	0.290
	Male (n = 24)	6.3 (0.7)	4.8–7.7	4.9 (5.0)	
	Female (n = 44)	7.5 (0.7)	6.1–8.8	6.0 (5.8)	
Vitamin B_1_ (mg/day)	All (n = 68)	1.5 (0.1)	1.3–1.7	1.3 (0.8)	0.608
	Male (n = 24)	1.6 (0.2)	1.3–2.0	1.4 (1.2)	
	Female (n = 44)	1.5 (0.1)	1.2–1.7	1.3 (0.7)	
Vitamin B_2_ (mg/day)	All (n = 68)	1.8 (0.1)	1.6–2.0	1.7 (1.0)	0.646
	Male (n = 24)	1.8 (0.2)	1.5–2.2	1.9 (1.1)	
	Female (n = 44)	1.7 (0.1)	1.5–2.0	1.6 (0.8)	
Vitamin B_6_ (mg/day)	All (n = 68)	1.6 (0.1)	1.5–1.8	1.5 (0.8)	0.092
	Male (n = 24)	1.8 (0.2)	1.5–2.1	1.8 (1.4)	
	Female (n = 44)	1.5 (0.1)	1.4–1.7	1.4 (0.5)	
Vitamin B_9_ (ug/day)	All (n = 68)	367 (20)	327–408	326 (180)	0.082
	Male (n = 24)	318 (28)	260–377	305 (152)	
	Female (n = 44)	392 (27)	338–446	361 (194)	
Vitamin B_12_ (ug/day)	All (n = 68)	4.0 (0.3)	3.5–4.5	3.3 (2.8)	0.074
	Male (n = 24)	4.6 (0.5)	3.6–5.6	3.9 (2.9)	
	Female (n = 44)	3.7 (0.3)	3.1–4.3	3.0 (2.4)	
Vitamin C (mg/day)	All (n = 68)	131 (10)	110–152	112 (121)	0.003
	Male (n = 24)	89 (15)	58–120	93 (88)	
	Female (n = 44)	154 (13)	127–180	126 (132)	
Sodium (mg/day)	All (n = 68)	2514 (148)	2219–2810	2267 (1629)	0.001
	Male (n = 24)	3314 (282)	2730–3897	3190 (1959)	
	Female (n = 44)	2101 (131)	1837–2365	1957 (1160)	
Potassium (mg/day)	All (n = 68)	2717 (105)	2507–2926	2720 (1167)	0.242
	Male (n = 24)	2549 (181)	2174–2924	2518 (1351)	
	Female (n = 44)	2808 (128)	2550–3067	2739 (946)	
Calcium (mg/day)	All (n = 68)	840 (45)	750–929	805 (428)	0.298
	Male (n = 24)	776 (80)	610–942	739 (652)	
	Female (n = 44)	881 (55)	771–992	825 (441)	
Iron (mg/day)	All (n = 68)	13.2 (0.6)	12.0–14.5	12.2 (6.2)	0.383
	Male (n = 24)	13.8 (1.0)	11.7–16.0	11.9 (5.5)	
	Female (n = 44)	12.9 (0.8)	11.3–14.4	12.6 (6.6)	
Zinc (mg/day)	All (n = 68)	8.7 (0.4)	7.9–9.6	8.3 (4.5)	0.781
	Male (n = 24)	8.9 (0.7)	7.4–10.4	8.9 (4.7)	
	Female (n = 44)	8.6 (0.5)	7.6–9.7	8.1 (3.5)	
Water (L/day)	All (n = 68)	1.9 (0.1)	1.6–2.1	1.7 (1.4)	0.332
	Male (n = 24)	1.7 (0.2)	1.3–2.1	1.5 (1.0)	
	Female (n = 44)	1.9 (0.1)	1.6–2.2	2.1 (1.3)	
Dietary Fiber (g/day)	All (n = 68)	18.8 (0.9)	16.8–20.8	16.9 (9.3)	0.014
	Male (n = 24)	15.6 (1.4)	12.7–18.5	15.3 (7.8)	
	Female (n = 44)	20.1 (1.1)	17.9–22.2	19.4 (11.1)	

CHO, digestible carbohydrates; CI, Confidence Interval; IQR, Interquartile range; MUFA, Monounsaturated Fatty acid. *p*-values show significant differences between the reported intakes from the self-identified male and self-identified female subjects.

**Table 3:** The status of physical activity and energy intake among the participants.

**Self-Identified Males**	**Sedentary**	**Low Active**	**Active**	**Very Active**
	n = 8 (33%)	n = 8 (33%)	n = 7 (30%)	n = 1 (4%)
Energy Intake (kcal/day)	2106 (154)	1786 (131)	2146 (285)	2312
95% CI	1742–2470	1463–2109	1449–2845	n/a
Median (IQR)	2091 (637)	1774 (583)	2275 (1433)	n/a
Calculated EER (kca/day)	2608	2617	2863	3139
EI/EER Ratio	0.81	0.68	0.75	0.74
**Self-identified Females**				
	n = 9 (21%)	n = 19 (43%)	n = 15 (34%)	n = 1 (2%)
Energy Intake (kcal/day)	1440 (143)	1792 (117)	1639 (107)	1595
95% CI	1111–1768	1545–2038	1545–2038	n/a
Median (IQR)	1333 (577)	1678 (464)	1500 (518)	n/a
Calculated EER (kca/day)	1878	2034	2136	3019
EI/EER Ratio	0.77	0.88	0.77	0.53

CI, Confidence Interval; IQR, Interquartile range; EI, Energy Intake; EER, Estimated Energy Requirement; n/a, not applicable.

## Data Availability

Data produced and analyzed through this manuscript will be available through a reasonable request made to the authors.

## References

[1] Riediger N.D., Moghadasian M.H. (2008). Patterns of fruit and vegetable consumption and the influence of sex, age and socio-demographic factors among Canadian elderly. J. Am. Coll. Nutr..

[2] Riediger N.D., Shooshtari S., Moghadasian M.H. (2007). The influence of sociodemographic factors on patterns of fruit and vegetable consumption in Canadian adolescents. J. Am. Diet. Assoc..

[3] Choi J. (2020). Impact of Stress Levels on Eating Behaviors among College Students. Nutrients.

[4] Mensah F.Z., Lane K.E., Richardson L.D. (2022). Determinants of eating behaviour in Black, Asian and Minority Ethnic (BAME) university students when living at and away from home: With a focus on the influence of food enculturation and food acculturation. Appetite.

[5] Yamamoto R., Tomi R., Shinzawa M., Yoshimura R., Ozaki S., Nakanishi K., Ide S., Nagatomo I., Nishida M., Yamauchi-Takihara K. (2021). Associations of Skipping Breakfast, Lunch, and Dinner with Weight Gain and Overweight/Obesity in University Students: A Retrospective Cohort Study. Nutrients.

[6] Sogari G., Velez-Argumedo C., Gómez M.I., Mora C. (2018). College Students and Eating Habits: A Study Using An Ecological Model for Healthy Behavior. Nutrients.

[7] Mauvais-Jarvis F., Merz N.B., Barnes P.J., Brinton R.D., Carrero J.J., DeMeo D.L., De Vries G.J., Epperson C.N., Govindan R., Klein S.L. (2020). Sex and gender: Modifiers of health, disease, and medicine. Lancet.

[8] Gualtierotti R. (2025). Bridging the gap: Time to integrate sex and gender differences into research and clinical practice for improved health outcomes. Eur. J. Intern. Med..

[9] Massey S.C., Whitmire P., Doyle T.E., Ippolito J.E., Mrugala M.M., Hu L.S., Canoll P., Anderson A.R., Wilson M.A., Fitzpatrick S.M. (2021). Sex differences in health and disease: A review of biological sex differences relevant to cancer with a spotlight on glioma. Cancer Lett..

[10] Francis J., Graubard B.I., Katki H., Jackson S.S. (2026). Sex and All-Cause Mortality in the US, 1999 to 2019. JAMA Netw. Open.

[11] Canadian Institutes of Health Research (2012). What a Difference Sex and Gender Make: A Gender, Sex and Health Research Casebook.

[12] Health Canada (2011). Eating Well with Canada’s Food Guide.

[13] Khawar H. (2018). Energy and Nutrient Intake Among University Students Enrolled in an Introductory Nutrition Course. Master’s Thesis.

[14] Canada H. Government of Canada. Canada.ca. https://www.canada.ca/en/health-canada/services/food-guide.html.

[15] Ree M., Riediger N., Moghadasian M.H. (2008). Factors affecting food selection in Canadian population. Eur. J. Clin. Nutr..

[16] Government of Canada IAP on RE (2023). Tri-Council Policy Statement: Ethical Conduct for Research Involving Humans—TCPS 2 (2022).

[17] Shiffler R.E. (1988). Maximum Z scores and outliers. Am. Stat..

[18] Frank G.C. (2008). Community Nutrition: Applying Epidemiology to Contemporary Practice.

[19] Graham J.W. (2009). Missing data analysis: Making it work in the real world. Annu. Rev. Psychol..

[20] Kim H.Y. (2013). Statistical notes for clinical researchers: Assessing normal distribution (2) using skewness and kurtosis. Restor. Dent. Endod..

[21] Hawkes C. (2013). Promoting Healthy Diets Through Nutrition Education and Changes in the Food Environment: An International Review of Actions and Their Effectiveness.

[22] Pem D., Jeewon R. (2015). Fruit and Vegetable Intake: Benefits and Progress of Nutrition Education Interventions- Narrative Review Article. Iran. J. Public Health.

[23] Wardle J., Parmenter K., Waller J. (2000). Nutrition knowledge and food intake. Appetite.

[24] Anding J.D., Suminski R.R., Boss L. (2001). Dietary intake, body mass index, exercise, and alcohol: Are college women following the dietary guidelines for Americans?. J. Am. Coll. Health.

[25] Korinth A., Schiess S., Westenhoefer J. (2010). Eating behaviour and eating disorders in students of nutrition sciences. Public Health Nutr..

[26] Ouellette C.D., Yang M., Wang Y., Yu C., Fernandez M.L., Rodriguez N.R., Chun O.K. (2012). Assessment of nutrient adequacy with supplement use in a sample of healthy college students. J. Am. Coll. Nutr..

[27] Wang D.D., Li Y., Bhupathiraju S.N., Rosner B.A., Sun Q., Giovannucci E.L., Rimm E.B., Manson J.E., Willett W.C., Stampfer M.J. (2021). Fruit and Vegetable Intake and Mortality: Results From 2 Prospective Cohort Studies of US Men and Women and a Meta-Analysis of 26 Cohort Studies. Circulation.

[28] Hu Y., Ding M., Sampson L., Willett W.C., Manson J.E., Wang M., Rosner B., Hu F.B., Sun Q. (2020). Intake of whole grain foods and risk of type 2 diabetes: Results from three prospective cohort studies. BMJ.

[29] Shivakoti R., Biggs M.L., Djoussé L., Durda P.J., Kizer J.R., Psaty B., Reiner A.P., Tracy R.P., Siscovick D., Mukamal K.J. (2022). Intake and Sources of Dietary Fiber, Inflammation, and Cardiovascular Disease in Older US Adults. JAMA Netw. Open.

[30] Yang J.J., Yu D., Xiang Y.B., Blot W., White E., Robien K., Sinha R., Park Y., Takata Y., Lazovich D. (2020). Association of Dietary Fiber and Yogurt Consumption with Lung Cancer Risk: A Pooled Analysis. JAMA Oncol..

[31] Gill S.K., Rossi M., Bajka B., Whelan K. (2021). Dietary fibre in gastrointestinal health and disease. Nat. Rev. Gastroenterol. Hepatol..

[32] Veettil S.K., Wong T.Y., Loo Y.S., Playdon M.C., Lai N.M., Giovannucci E.L., Chaiyakunapruk N. (2021). Role of Diet in Colorectal Cancer Incidence: Umbrella Review of Meta-analyses of Prospective Observational Studies. JAMA Netw. Open.

[33] Van Blarigan E.L., Ou F.S., Bainter T.M., Fuchs C.S., Niedzwiecki D., Zhang S., Saltz L.B., Mayer R.J., Hantel A., Benson A.B. (2022). Associations Between Unprocessed Red Meat and Processed Meat with Risk of Recurrence and Mortality in Patients with Stage III Colon Cancer. JAMA Netw. Open.

[34] Kappeler R., Eichholzer M., Rohrmann S. (2013). Meat consumption and diet quality and mortality in NHANES III. Eur. J. Clin. Nutr..

[35] U.S. Department of Agriculture (2020). Dietary Guidelines for Americans 2020–2025.

[36] Ahvanooei M.R.R., Norouzian M.A., Vahmani P. (2022). Beneficial Effects of Vitamins, Minerals, and Bioactive Peptides on Strengthening the Immune System Against COVID-19 and the Role of Cow’s Milk in the Supply of These Nutrients. Biol. Trace Elem. Res..

[37] Bonjour J.P., Kraenzlin M., Levasseur R., Warren M., Whiting S. (2013). Dairy in adulthood: From foods to nutrient interactions on bone and skeletal muscle health. J. Am. Coll. Nutr..

[38] Bedard D., Shatenstein B., Nadon S. (2004). Underreporting of energy intake from a self-administered food-frequency questionnaire completed by adults in Montreal. Public Health Nutr..

[39] Goldberg G.R., Black A.E., Jebb S.A., Cole T.J., Murgatroyd P.R., Coward W.A., Prentice A.M. (1991). Critical evaluation of energy intake data using fundamental principles of energy physiology: 1. Derivation of cut-off limits to identify under-recording. Eur. J. Clin. Nutr..

[40] Muñoz-Yáñez C., Molina-Flores C.A., Guandorena-Gómez J.O. (2024). Determinación de la infradeclaración de la ingesta de energía por el método de Goldberg y Black en la cohorte FACSA. Estudio piloto [Determination of the missrreporting of energy intake by Goldberg and Black in the FACSA cohort. Pilot study]. Nutr. Hosp..

[41] Black A.E. (2000). The sensitivity and specificity of the Goldberg cut-off for EI:BMR for identifying diet reports of poor validity. Eur. J. Clin. Nutr..

[42] Kljajević V., Stanković M., Đorđević D., Trkulja-Petković D., Jovanović R., Plazibat K., Oršolić M., Čurić M., Sporiš G. (2021). Physical Activity and Physical Fitness among University Students-A Systematic Review. Int. J. Environ. Res. Public Health.

[43] Khurshid S., Al-Alusi M.A., Churchill T.W., Guseh J.S., Ellinor P.T. (2023). Accelerometer-Derived “Weekend Warrior” Physical Activity and Incident Cardiovascular Disease. JAMA.

[44] Herbert C. (2022). Enhancing Mental Health, Well-Being and Active Lifestyles of University Students by Means of Physical Activity and Exercise Research Programs. Front. Public Health.

[45] Physical Activity Guidelines for American. https://odphp.health.gov/our-work/nutrition-physical-activity/physical-activity-guidelines.

[46] Pate R.R., Pratt M., Blair S.N., Haskell W.L., Macera C.A., Bouchard C., Buchner D., Ettinger W., Heath G.W., King A.C. (1995). Physical activity and public health. A recommendation from the Centers for Disease Control and Prevention and the American College of Sports Medicine. JAMA.

[47] (2013). Canadian Physical Activity, and Sedentary Behaviour Guidelines. Canadian Society for Exercise Physiology-Physical Activity Training for Health (CSEP-Path).

[48] Directorate-General for Health and Food Safety (2008). EU Physical Activity Guidelines.

[49] Swinburn B.A., Kraak V.I., Allender S., Atkins V.J., Baker P.I., Bogard J.R., Brinsden H., Calvillo A., De Schutter O., Devarajan R. (2019). The Global Syndemic of Obesity, Undernutrition, and Climate Change: The Lancet Commission report. Lancet.

